# Plexin B3 promotes neurite outgrowth, interacts homophilically, and interacts with Rin

**DOI:** 10.1186/1471-2202-6-53

**Published:** 2005-08-25

**Authors:** Christine Hartwig, Andres Veske, Sarka Krejcova, Georg Rosenberger, Ulrich Finckh

**Affiliations:** 1 Institut für Humangenetik, Universitätsklinikum Hamburg-Eppendorf, Universität Hamburg, Hamburg, Germany; 2 Institute of Gene Technology, Tallinn Technical University, Tallinn, Estonia; 3 Laboratoriumsmedizin Dortmund, Dortmund, Germany

## Abstract

**Background:**

Plexins, known to date as receptors of semaphorins, are implicated in semaphorin-mediated axon repulsion and growth cone collapse. However, subtype-specific functions of the majority of the nine members of the mammalian plexin family are largely unknown. In order to investigate functional properties of B-plexins, we analyzed the expression of human and murine plexin B3 and expressed full-length human plexins B2 (B2) and B3 (B3) in NIH-3T3 cells.

**Results:**

Unexpectedly, B3 strongly and B2 moderately stimulate neurite outgrowth of primary murine cerebellar neurons. Both plexins mediate Ca^2+^/Mg^2+^-dependent cell aggregation due to homophilic *trans*-interaction, which is strong in the case of B3 and moderate for B2. Using different deletion constructs we show that the sema domain of B3 is essential for homophilic interaction. Using yeast two-hybrid analysis, we identified the neuron-specific and calmodulin-binding Ras-related GTPase Rin as an interaction partner of the intracellular part of B3, but not of B2. Rin, also known for its neurite outgrowth-inducing characteristics, co-localizes and co-immunoprecipitates with B3 in co-transfected COS-7 cells.

**Conclusion:**

Our data suggest an involvement of homophilic interaction of B3 in semaphorin-independent signaling mechanisms positively influencing neuronal morphogenesis or function. Furthermore the neuron-specific small GTPase Rin is involved in downstream signaling of plexin B3.

## Background

During the development of the nervous system neurons respond to attractive and repulsive guidance cues to navigate to their final targets [1,2]. The nine mammalian plexins, A1–4, B1–3, C1, and D1 [3,4] are characterized by a sema domain, three cysteine-rich repeats (MRS, Met-related sequences, or PSI, plexins, semaphorins, and integrins), three glycine/proline-rich repeats (IPT, immunoglobulin-like fold shared by plexins and transcription factors), a single-pass transmembrane region, and an intracellular SP (sex plexin) domain consisting of two different parts [5]. Plexins are known as semaphorin receptors [6]. Molecules associated with plexins in receptor complexes include cell adhesion molecule L1, the scatter factor receptors Met and Ron, erbB-2, OTK, and VEGFR2 [7-14]. Interactions have been shown between plexin C1 and semaphorin 7A [3,15], plexin D1 and semaphorin 3E [16], plexin B1 and semaphorin 4D [3], and plexin B3 and semaphorin 5A [17]. Semaphorin 5A induces growth cone collapse in retinal ganglion cells, has axon-repelling activity [18], induces cellular collapse, and leads to inhibition of integrin-based adhesion of NIH-3T3 fibroblasts expressing recombinant plexin B3 [17]. The cytoplasmic C-terminus of B plexins activates Rho GTPase through Rho guanine nucleotide exchange factors PDZ-RhoGEF and LARG [19-24]. Based on this C-terminal interaction, plexin B1 mediates semaphorin 4D-induced growth cone collapse in neurons [20]. Independently of this mechanism, a direct down regulation of the activity of neurite outgrowth-promoting GTPase R-Ras by the GTPase activating protein (GAP)-homologous domain of plexin B1 has been shown [25]. Thus, according to published data, plexins appear to be mainly involved in the repulsive activities of semaphorins on neuronal cells.

We found evidence for plexin B3- and B2-dependent stimulation of neurite outgrowth, subtype-specific homophilic interaction of B3 and B2, respectively, and an interaction of B3 with neuron-specific GTPase Rin, the latter one known for its involvement in neurite outgrowth.

## Results

### Expression and alternative splicing of PLXNB3

**Figure 1:**
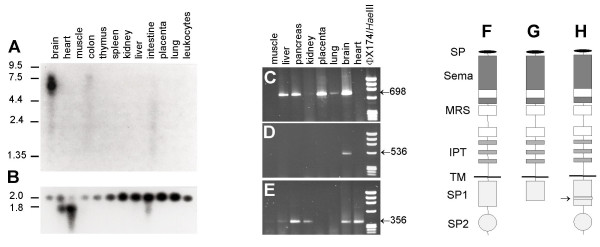
**Expression and alternative splicing of PLXNB3 in adult human tissues**. **(A)**, expression analysis of *PLXNB3 *in adult human tissues by poly(A)^+ ^mRNA northern blot. **(B)**, the blot was stripped and reprobed with β-actin probe. Molecular weight in kb is indicated at the left side. **(C-E)**, tissue distribution of three isoforms of *PLXNB3 *due to alternative splicing of the 3'- part of exon 27. Fragments were amplified by RT-PCR using a common forward primer and isoform-specific reverse primers. Fragment sizes (bp) are given on the right. ΦX174 DNA cleaved by *Hae*III was used as size standard. **(C)**, 698 bp fragment containing full length exon 27. **(D)**, 536 bp fragment lacking the 3'-terminal part of exon 27 and coding for a C-terminally truncated B3. **(E)**, 356 bp fragment lacking (in-frame) 246 bp of exon 27. **(F-H)**, possible protein structures of B3 predicted by mRNA isoforms generated through alternative splicing of exon 27. **(F)**, full length isoform; **(G)**, C-terminally truncated B3 predicted by the isoform shown in D; **(H)**, structure of the B3 isoform lacking 246 bp of exon 27 (missing 83 amino acids marked by arrow) as shown in E. SP, signal peptide; Sema, semaphorin domain; MRS, Met-related sequences; IPT, immunoglobulin-like fold shared by plexins and transcription factors; TM, transmembrane domain; SP1/SP2, two different parts of the sex plexin domain.

Northern blot analysis of 12 different human organs (Figure 1A) revealed a strong band of ~6.2 kb from the brain sample but not the remaining organs, indicating that *PLXNB3 *is expressed abundantly only in brain. The estimated size of the mRNA corresponds well with that of the mature message predicted from the cloned full-length human cDNA [GenBank:AF149019]. BLASTn screening of human dbEST by AF149019 revealed 56 fully matching entries, all of them representing the 3'-end of the transcript and two variants. EST [GenBank:BF345653] from oligodendroglioma lacks 246 nucleotides of exon 27, corresponding to bp 4,595–4,840 of AF149019. This gap predicts an in-frame loss of 82 codons (aa 1,495–1,575). EST [GenBank:H51489] from adult brain lacks the 67 3'-terminal nucleotides of exon 27 (bp 4,774–4,840 of AF149019). This gap predicts a C-terminally truncated isoform of B3 due to a frame-shift resulting in the inclusion of nine amino acids (aa 1,554–1,563) followed by a premature stop. These findings suggest alternative splicing and the existence of at least three different B3-isoforms due to skipping of various parts of exon 27. Differential expression of the three isoforms in human organs was confirmed by PCR using isoform-specific primers. As shown in Figure 1C the full-length exon 27-isoform was detectable in the majority of the organs analyzed but skeletal muscle and heart. cDNA of the truncated isoform was detectable only in the brain (Figure 1D), whereas the isoform lacking 82 codons was present in skeletal muscle, liver, pancreas, kidney, brain, and heart (Figure 1E). The structures of full length B3 and the two different isoforms are shown in figure 1F–H.

### Analysis of recombinantly expressed and cerebral plexin B3 protein

**Figure 2:**
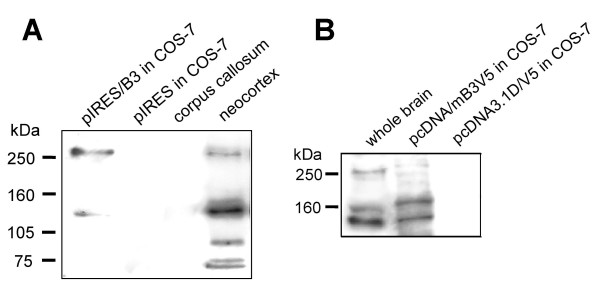
**Detection of plexin B3 protein in human and murine brain**. **(A) **Western blot detection of human plexin B3 (B3) protein in pIRES/B3-tranfected COS-7 cells (lane 1) and human brain (corpus callosum, lane 3; neocortex, lane 4) using antibody pAbB3-B. COS-7 cells transfected with empty pIRES served as negative control (lane 2). **(B) **Western blot detection of B3 protein in mouse brain (lane 1) and of C-terminally truncated recombinant mouse B3 in pcDNA/mB3V5-transfected COS-7 cells (lane 2) using antibody pAbmB3. COS-7 cells transfected with empty pcDNA3.1D/V5-His served as negative control (lane 3).

**Figure 3:**
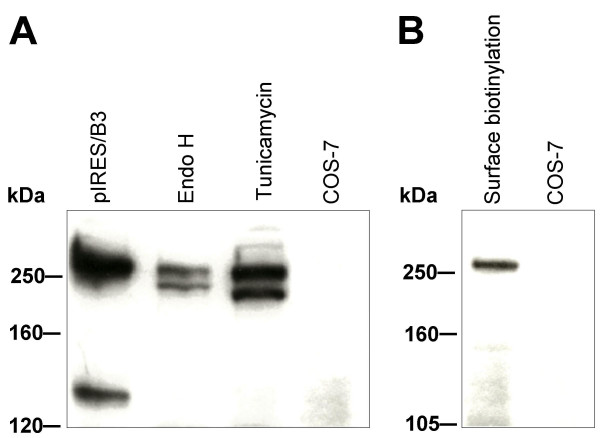
**Glycosylation analysis and cell surface protein biotinylation of plexin B3**. **(A) **COS-7 cells were transfected with pIRES/B3 and lysed. Untreated lysate served as positive control (B3 pIRES). To analyze cell surface expression and glycosylation of B3 total cell lysates were treated with deglycosylating enzyme Endo H (EndoH). Alternatively, growing cells were incubated by co-translational glycosylation-inhibitor tunicamycin (Tunicamycin). Non-transfected COS-7 cells served as negative control. Lysates were analyzed by Western blot using B3-specific antibody pAbB3-B. **(B) **After biotinylation of cell surface proteins of cells transfected with pIRES/B3 and immunoprecipitation with pAbB3-B, B3 located on the cell surface was detected in Western blot with alkaline phosphatase-conjugated streptavidin (Surface biotinylation). Non-transfected cells treated as described before served as negative control (COS-7).

**Figure 4:**
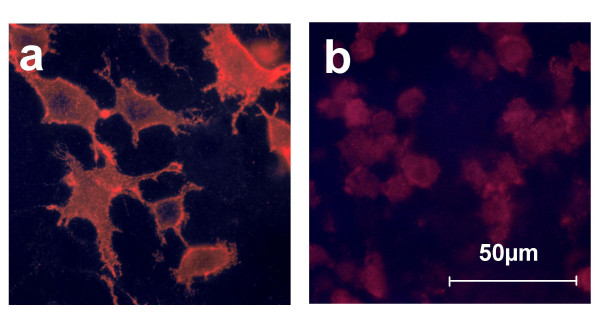
**Cell surface localization of recombinant plexin B3 protein**. **(a) **Immunocytochemistry using antibody pAbB3-B was performed on living NIH-3T3 cells stably transfected with pIRES/B3 encoding full-length B3. Cy3 conjugated goat anti-rabbit IgG was used as secondary antibody. **(b) **NIH 3T3 cells transfected with empty pIRES and treated as described for a.

Conceptual translation of PLXNB3 cDNA [GenBank: AF149019] predicts a protein of 1,909 aa with a molecular mass of 207 kDa and an isoelectric point of 6.21. Western blot (WB) analysis of proteins from COS-7 cells stably overexpressing full-length B3 and from human neocortex (but not from corpus callosum) using antibody pAbB3-B against the third IPT-domain of human B3 revealed bands of ~260 kDa and ~140 kDa that were absent in nontransfected control cells (Figure 2A). Antibody pAbB3-A against the human sema domain of B3 only detected the 260 kDa band (data not shown). Taken together, these data suggest proteolytic processing of the extracellular portion of B3 similar to that described for plexins B1 and B2 [26]. Within the extracellular domains of B3 flanked by the epitopes of pAbB3-A and pAbB3-B there are two RXXR sites corresponding to the minimal consensus motif required by proprotein convertases. Cleavage at one of these sites would remove the sema domain leaving the truncated transmembrane part of B3 not recognizable by pAbB3-A. pAbB3-B did not cross-react with plexins B1, B2, or A1 expressed in COS-7 cells and was therefore also useful for the analysis of co-immunoprecipitation of B3 with these molecules (see figure 12B–D). Mouse B3 was detected by WB analysis of adult murine brain lysate using antibody pAbmB3 against the sema domain of mouse B3 (Figure 2B, first lane). pAbmB3 detected bands of ~260 kDa, ~160 kDa, and ~140 kDa. This suggests posttranslational processing of murine B3 similar or analogous to that of human B3. Minor differences in band patterns between the murine and the human WB may be due to the different epitopes recognized by the antibodies. pABmB3 also recognized C-terminally truncated mouse B3 expressed in pcDNA/mB3V5-transfected COS-7 cells used as positive control (Figure 2B, lane 2). B3 has ten potential N-glycosylation sites (Asn-X-Ser/Thr, X≠Pro). The 260 kDa protein detected by pAbB3-B in human brain (Figure 3A, lane 1) was resistant to EndoH treatment (Figure 3A, lane 2) yielding a distinct band of ~260 kDa in addition to a novel band running at ~200–230 kDa. The latter one suggests the presence of incompletely processed B3 non-resistant to EndoH in the transfected cells. Treatment with tunicamycin resulted in a relatively reduced intensity of the 260 kDa band and the appearance of a novel band of ~200 kDa. These data suggest that the 200 kDa band corresponds to deglycosylated full-length B3, whereas the 260 kDa band represents fully glycosylated, mature, full-length transmembraneous B3 supposed to be located at the cell surface. This was confirmed by exclusive detection of a sharp band of a biotinylated 260 kDa protein after pAbB3-B-immunoprecipitation of surface-biotinylated protein of stably transfected COS-7 cells (Figure 3B, first lane). As shown by immunocytochemistry of living cells stably expressing full-length B3 (Figure 4a), a significant proportion of B3 is detectable at the cell surface.

### B3 stimulates neurite outgrowth

**Figure 5:**
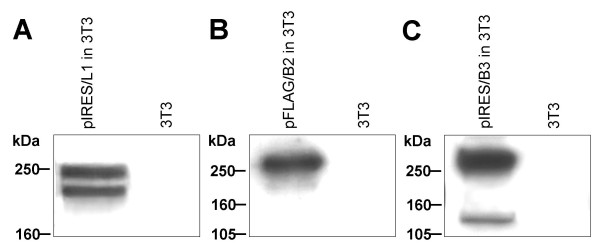
**Expression of plexin B2, plexin B3 and L1 protein in stably transfected NIH-3T3 cells**. Western blot detection of human L1, plexin B2 and plexin B3 protein in stably transfected NIH-3T3 substrate cells. In all cases same protein amounts were applied and non-transfected NIH-3T3 cells served as negative control. **(A) **Western blot detection of human L1 protein in stably pIRES/L1-tranfected NIH-3T3 cells using antibody pAbex2. **(B) **Western blot detection of human plexin B2 (B2) protein in stably pFLAG/B2-tranfected NIH-3T3 cells using anti-FLAG antibody. **(C) **Western blot detection of human plexin B3 (B3) protein in stably pIRES/B3-tranfected NIH-3T3 cells using antibody pAbB3-B.

**Figure 6:**
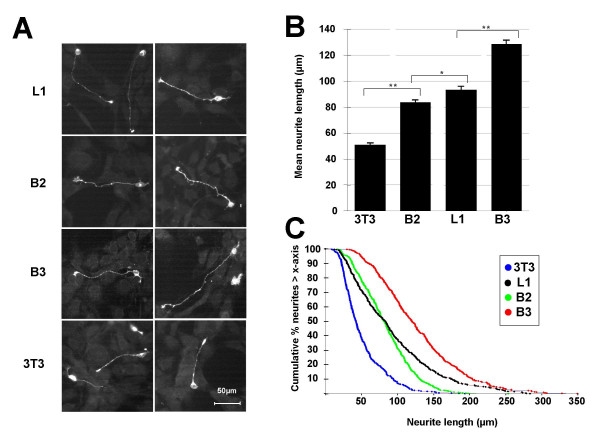
**Plexins B2 and B3 stimulate neurite outgrowth**. **(A) **Examples of primary murine cerebellar neurons isolated from six days old c57BL/6J mice and grown on L1-, B2-, or B3- expressing transfected (L1, B2, B3) or non-transfected (3T3) NIH-3T3 substrate cells. **(B) **Mean length of neurites of murine cerebellar neurons grown for 24 h on NIH-3T3 substrate cells. Nontransfected substrate cells (3T3) served as negative control. Cells transfected with pIRES/L1 encoding neuronal cell surface molecule L1 (L1) served as positive control. The strongest stimulation of neurite outgrowth was observed on substrate cells transfected with pIRES/B3 encoding full-length plexin B3 (B3). Plexin B2-expressing substrate cells (B2) were transfected with expression construct pFLAG/B2. Each outgrowth assay was done in three independent experiments. Pooled data of the triple experiments are shown. A minimum of 400 neurons were analyzed for each substrate cell type. Error bars: S.E. of mean × 1. Mean neurite length differed significantly between all groups (ANOVA; **, p < 0.0005; *, p = 0.0033). **(C) **Culmulative size distribution patterns of the neurites given in B.

We analyzed the effects of recombinant B2 and B3 expressed by NIH-3T3 substrate cells on neurite outgrowth of cerebellar neurons of six days old c57BL/6J mice. Polyclonal NIH-3T3 cells stably expressing recombinant human L1 [27,28] (Figure 5A) served as a positive control substrate. Polyclonal NIH-3T3 cells, negative for *PLXNB2 *and *PLXNB3 *cDNA in RT-PCR (data not shown), express at comparable levels recombinant human B2 or B3 at the cell surface after stable transfection using the respective full-length expression constructs pFLAG/B2 or pIRES/B3 (Figure 5B–C). Cells expressing B3 displayed increased cell adhesive properties to surfaces and a more flattened cell shape. No such changes were observed in the cells expressing recombinant B2. Non-transfected NIH-3T3 cells, known to stimulate neurite outgrowth only moderately [28], were used as negative substrate control. As suggested by the examples shown in Figure 6A and shown in Figure 6B and 6C, both plexins stimulate neurite outgrowth. Mean outgrowth stimulation through B3-positive substrate cells was significantly higher than that through L1 that in turn showed a higher stimulation than B2. Mean neurite length differed significantly between all groups (ANOVA p ≤ 0.0033). Mean length (± S.D.) of neurites of neurons grown on nontransfected cells was 51 μm ± 30; mean neurite length of neurons grown on cells expressing B2, L1, or B3 was 84 μm ± 37, 93 μm ± 59, and 129 μm ± 58. The neurites of the outgrowth experiments revealed similarly shaped size distribution curves (Figure 6C).

### Ca^2+^/Mg^2+^-dependent B3-homophilic interaction in trans promotes cell adhesion

**Figure 7:**
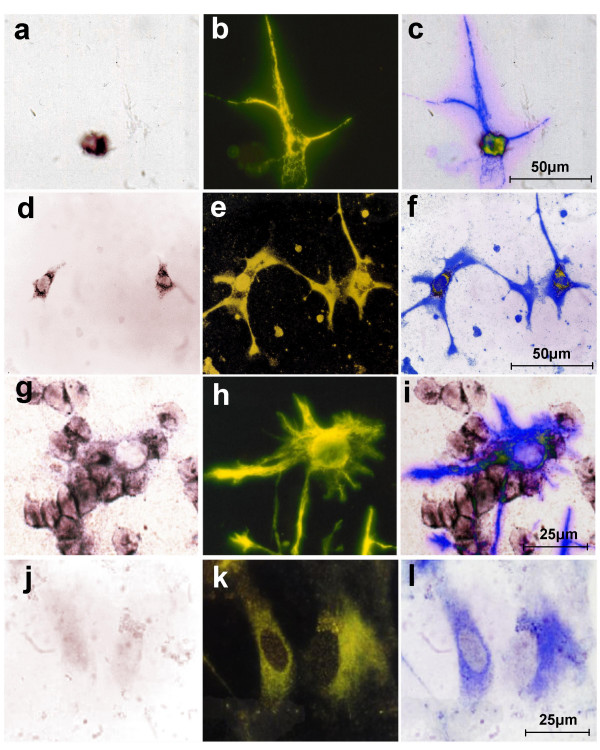
**Expression of *PlxnB3 *in cultivated primary murine cerebellar neurons**. Expression of *PlxnB3 *in cultivated primary cerebellar neurons isolated from six days old c57BL/6J mice (**a-f**; 400 × magnification) but not in astrocytes **(g-i) **or oligodendrocytes **(j-l) **(630 × magnification). The cells were cultivated from a cerebellar homogenate of a six days old mouse and analyzed by combined *in situ *hybridization (ISH) using a *PlxnB3*-specific probe **(a, d, g, j) **and immunocytochemistry (IC) using cell type-specific antibodies **(b, e, h, k)**. Overlays of ISH bright field signals and IC signals (blue) are shown in **c, f, i, **and **l**. The cells were grown under the respective selective conditions for neurons, astrocytes or oligodendrocytes. Following ISH slides were used for IC using primary antibodies against Neuromodulin **(b) **or NeuN **(e) **for neurons, GFAP for astrocytes **(h) **or CNPase for Oligodendrocytes **(k)**. The Neuromodulin- and NeuN-positive cells (neurons) shown in **b **and **e **are positive for *PlxnB3 *mRNA **(a, d)**.

**Figure 8:**
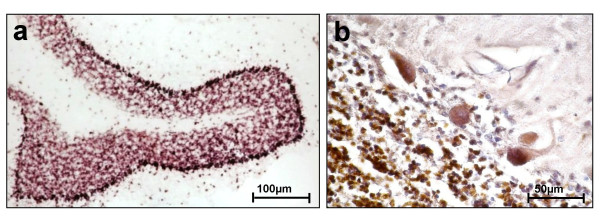
**Neuronal expression of B3 in adult murine and human cerebellum**. **(a) **Non-radioactive *in situ *hybridization of *PlxnB3 *mRNA in adult murine cerebellum using a probe corresponding to nucleotides 4,647 – 5,936 of *PlxnB3 *cDNA. Purkinje and granular cells show most intense staining (200 × magnification). **(b) **Detection of plexin B3 protein in adult human cerebellum by immunohistochemistry using antibody pAbB3-B. Positive signal is represented by brown staining and most intense in cerebellar Purkinje cells and neurons of the granular layer (400 × magnification).

Primary cerebellar neurons (Figure 7a–f), but not astrocytes (Figure 7g–i) or oligodendrocytes (Figure 7j–l) of six days old mice grown under selective conditions express *PlxnB3*, suggesting the possibility that neuronally expressed B3 may be involved in mediation of the B3-dependent stimulation of neurite outgrowth. To determine the *in vivo *expression of *PlxnB3*, we performed *in situ *hybridization (ISH) in adult mouse cerebellum using two different probes covering nucleotides 4,647 – 5,936 (Figure 8a) or 3,744 – 5,679 like the probe used by Worzfeld et al. [29] (data not shown). With both probes, strongest labeling was observed in cerebellar neurons, i.e. Purkinje and granular cells. Immunohistochemistry (IHC) of adult human cerebellum using antibody pAbB3-B also revealed staining of Purkinje and granular cells (Figure 8b). Furthermore, murine and human B3 co-immunoprecipitate (see next paragraph). This allowed us to hypothesize that homophilic interaction of B3 and possibly also that of B2 may underlie the B3- and B2-specific stimulation of neurite outgrowth, respectively.

**Figure 9:**
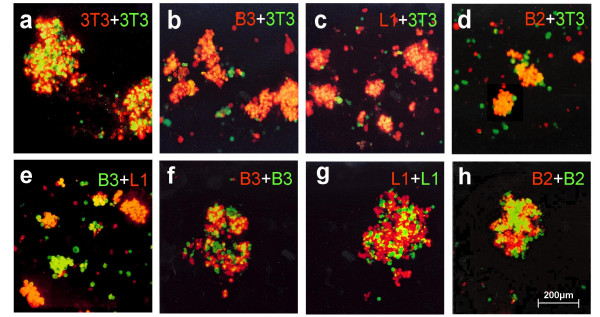
**Cell aggregation mediated by subtype-specific homophilic interaction *in trans *of plexins B2 and B3**. Aggregation of NIH-3T3 cells expressing full-length recombinant B3, L1, or B2 and non-transfected cells (100 × magnification). **(a) **Negative control non-transfected cells labeled with DiO (green) were mixed 1:1 with DiI-labeled ones (red) and incubated in DMEM for 45 min prior to fluorescence microscopy. The formed aggregates contain approximately equal proportions of DiO- and DiI-labeled cells. **(b)-(d) **Non-transfected cells labeled with DiO and DiI-labeled cells stably expressing full-length B3 **(b)**, L1 **(c)**, or B2 **(d) **were mixed 1:1, respectively and treated as described in a. Predominance of DiI-labeled cells in the formed aggregates indicates cell-cell-adhesion due to homophilic interaction in *trans *mediated by B3, L1, and B2. **(e) **L1-transfected cells labeled with DiI and DiO-labeled cells stably expressing B3 were mixed 1:1. The almost pure red or pure green aggregates indicate preferential homotypic interactions of both B3 and L1, respectively. **(f-h) **As additional negative control cells labeled with DiO and expressing B3 (f), L1 (g) or B2 (h) were mixed 1:1 with DiO labeled cells expressing B3 (f), L1 (g) or B2 (h). The formed aggregates contain approximately equal proportions of DiO- and DiI-labeled cells.

In order to investigate the possibility of homophilic interaction, we performed cell aggregation assays using transfected NIH-3T3 cells stably expressing B3 or B2. Transfected cells expressing L1 and non-transfected cells served as positive and negative controls, respectively. B3-, B2-, and L1-expressing cells were stained with DiI (red) and mixed 1:1 with non-transfected cells stained with DiO (green). Aggregates of non-transfected DiI-stained cells mixed 1:1 with DiO-stained ones were composed of approximately equal proportions of DiI- and DiO-labeled cells (Figure 9a). In contrast, there was strong predominance of DiI-stained B3-expressing cells (red) in the aggregates as shown in Figure 9b, indicating an enhanced aggregation due to homophilic interaction of B3 in *trans*. Similar results were obtained with cells expressing L1 (Figure 9c) and to a smaller extent also for those expressing B2 (Figure 9d). Furthermore, DiI-stained L1-expressing cells (red) were mixed 1:1 with DiO-stained B3-expressing ones (green); the almost monochrome green and red aggregates indicate specific and preferential homotypic interactions of both L1 and B3 (Figure 9e), respectively. As further negative control for the aggregation assay cells expressing B3 (Figure 9f), L1 (Figure 9g) or B2 (Figure 9h) labeled alternatively with DiO or DiI were mixed 1:1, respectively; all aggregates were composed of approximately equal proportions of DiI- and DiO-labeled cells.

**Figure 10:**
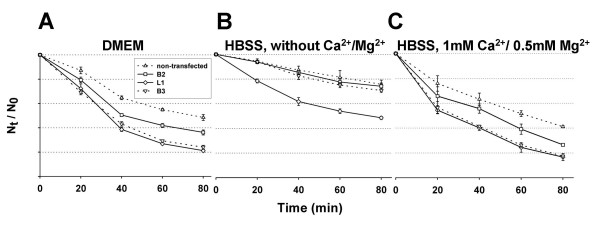
**Plexins B2 and B3 promote Ca^2+^/Mg^2+^-dependent cell aggregation**. Cells stably expressing the neuronal cell adhesion molecule L1 known to promote Ca^2+^-independent cell aggregation were used as positive control. Time-dependent decrease of aggregation index N_t_/N_0 _indicates decreasing total number of particles due to an increasing proportion of cells aggregated. Given are mean values of three independent experiments with error bars = S.E. of mean × 1. Aggregation assays were performed in DMEM **(A)**, HBSS without Ca^2+^/Mg^2+ ^**(B)**, and in HBSS with 1 mM Ca^2+^/0,5 mM Mg^2+ ^**(C)**.

Time dependent aggregation of cells expressing B2 was moderately enhanced, whereas that of cells expressing B3 or L1 was strongly enhanced (Figure 10). After 80 min aggregation time the number of particles decreased by 79%, 76%, 64%, and 52% in L1-, B3-, B2, and non-transfected cells, respectively (Figure 10A). As shown in Figures 10B and 10C, absence of divalent cations abolishes completely both B2- and B3-dependent but not L1-dependent aggregation, the latter one known to be independent of divalent cations [30].

### Homophilic interaction of B3 is mediated by the sema domain

**Figure 11:**
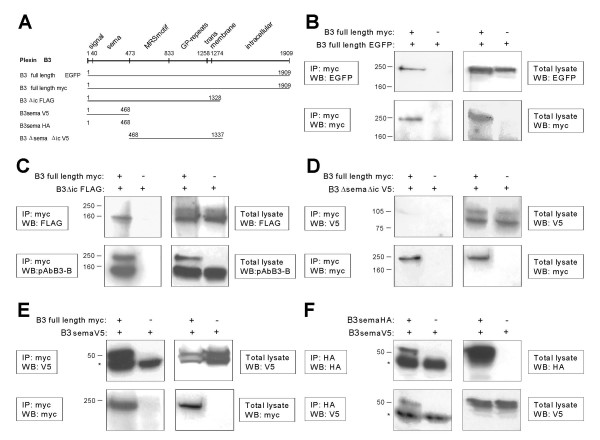
**Co-immunoprecipitation experiments showing homophilic interaction of B3 mediated by the sema domain**. COS-7 cells were co-transfected with various full-length and deletion constructs of B3. Cells transfected with a putative interaction partner and the corresponding/respective vector lacking an insert served as negative controls. Cells were lysed and immunoprecipitations (IP) were performed by various antibodies. Total lysates and IPs were analyzed by Western blot (WB) using antibodies as indicated in the figures. Bands marked with * represent antibodies precipitated by protein-A-agarose. **(A) **Human B3 constructs used for the IP experiments. **(B) **Cells were co-transfected with pSecTag2B/B3 encoding myc-tagged full-length B3 and pEGFP-N1/B3 encoding EGFP-tagged full-length B3. IP was performed with anti-myc antibody and shows homophilic interaction of B3. **(C-E) **Co-IP of three different B3 deletion mutants with anti-myc antibody against myc-tagged full-length B3, demonstrating homophilic interaction mediated through the sema domain. Cells were co-transfected with pSecTag2B/B3 and pFLAG/B3Δic encoding B3 missing the intracellular part **(C)**, pcDNA3.1/B3ΔsemaΔic encoding a V5-tagged fragment of B3 missing the intracellular part and the sema domain **(D)**, or pcDNA3.1/B3sema encoding the V5-tagged sema domain of B3 **(E)**. **(F) **Cells were co-transfected with pcDNA3.1/B3sema encoding V5-tagged sema domain of B3 and with pcDNA3.1/B3semaHA encoding HA-tagged sema domain of B3. Co-IP was performed using anti-HA antibody and suggests that the sema domain is essential and sufficient for homophilic binding.

**Figure 12:**
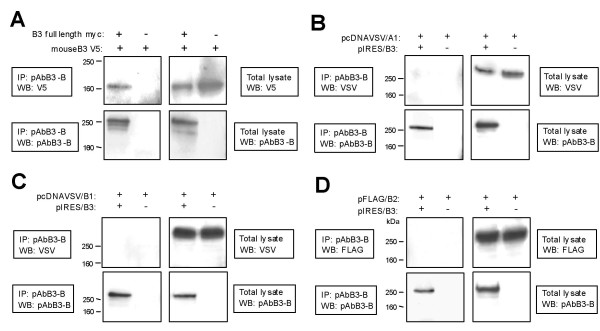
**Co-immunoprecipitation (IP) experiments showing homophilic interaction of human and murine B3 and no interaction of B3 with plexins A1, B1 or B2**. Total lysates and IPs were analyzed by Western blot (WB) using antibodies as indicated in the figures. IP was performed with pAbB3-B against human B3 and shows interaction between mouse and human B3 and no interaction between B3 and human plexins A1, B1 and B2. **(A) **Cells were co-transfected with pSecTag2B/B3 encoding myc-tagged human full-length B3 and pcDNA3.1/mB3 encoding V5-tagged mouse B3 lacking most of its intracellular part. Cells transfected with pcDNA3.1/mB3 and pSecTag2B vector without insert served as negative control. **(B) **COS-7 cells were co-transfected with pIRES/B3 encoding non-tagged full-length human B3 and pcDNAVSV/A1 encoding VSV-tagged full-length human plexin A1. Cells co-transfected with pcDNAVSV/A1 and pIRES vector without insert served as negative control. **(C) **COS-7 cells were co-transfected with pIRES/B3 and pcDNAVSV/B1 encoding VSV-tagged full-length human plexin B1. Cells co-transfected with pcDNAVSV/B1 and pIRES vector without insert served as negative control. **(D) **COS-7 cells were co-transfected with pIRES/B3 and pFLAG/B2 encoding FLAG-tagged full-length human plexin B2. Cells co-transfected with pFLAG/B2 and pIRES vector without insert served as negative control.

Homophilic binding of B3 was further analyzed by co-immunoprecipitation (IP) of full-length B3 and several deletion mutants (Figure 11A). Homophilic binding of full-length B3 was shown by anti-myc IP of lysates of cells co-transfected with expression constructs pEGFP-N1/B3 encoding EGFP-tagged and pSecTag2B/B3 encoding myc-tagged full-length B3, followed by detection with anti-EGFP antibodies (Figure 11B). Full-length B3 co-immunoprecipitates with deletion mutants containing the sema domain alone or lacking the intracytoplasmic part of B3 (B3Δic FLAG) but not with those lacking both the sema domain and the intracytoplasmic part (B3ΔsemaΔic V5; Figure 11C–E). In addition, V5-tagged sema domain (B3sema V5) co-immunoprecipitates with HA-tagged sema domain (B3sema HA; Figure 11F). These data indicate that the sema domain is both essential and sufficient for homophilic binding. Similarly, full-length human B3 (and B3Δic FLAG but not B3ΔsemaΔic V5, data not shown) co-immunoprecipitates with mouse B3 lacking most of its intracellular part (Figure 12A). This supports the assumption that a quasi homophilic interaction of human and murine B3 in *trans *may underlie the observed B3-dependent stimulation of neurite outgrowth. Full-length B3 co-immunoprecipitated with its known [17] ligand semaphorin 5A (data not shown); IP of lysates of cells co-expressing B3 and plexins A1, B1, or B2 revealed no evidence for heterophilic interaction of B3 with these molecules (Figure 12B–D).

### Rin, an intracellular interaction partner of B3

**Figure 13:**
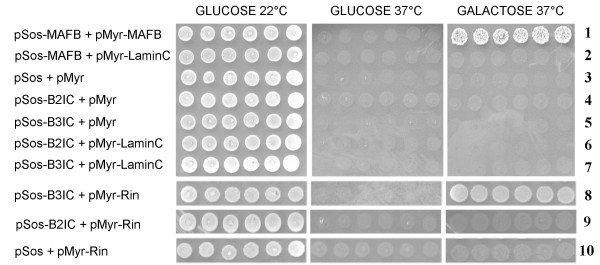
**Plexin B3 interacts with Rin in the Sos recruitment system**. Various pMyr and pSos plasmid combinations (as indicated on the left) were used for co-transformation of cdc25H yeast cells representing a positive control (line 1) and negative controls (lines 2–7, 10). Six independent transformants were spotted and grown for six days on glucose medium at 22°C (left panel) or 37°C (middle panel), and on galactose medium at 37°C (right panel). Cdc25H cells re-transformed with pSos-B3IC and pMyr-Rin (line 8) grow on galactose medium at 37°C for six days (right panel), showing interaction between full length human Rin and the intracellular part of human B3 in this system. Cells re-transformed with pSos-B2IC and pMyr-Rin (line 9) show no growth on galactose medium at 37°C after six days (right panel), demonstrating that the intracellular part of human B2 and human Rin do not interact in this system. Rin co-transfected with pSos-vector without insert is not able to induce growth on galactose at 37°C (line 10).

**Figure 14:**
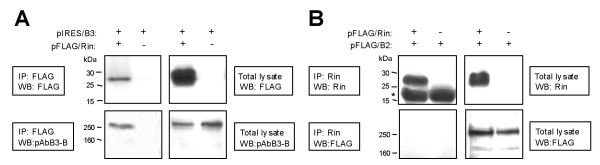
**Co-immunoprecipitation (IP) experiments showing interaction of B3 with Rin**. Total lysates and IPs were analyzed by Western blot (WB) using antibodies as indicated in the figures. **(A) **COS-7 cells were co-transfected with pIRES/B3 encoding non-tagged full-length human B3 and pFLAG/Rin encoding FLAG-tagged full-length human Rin. Cells co-transfected with pIRES/B3 and pFLAG vector without insert served as negative control. IP was performed using anti-FLAG antibodies. **(B) **COS-7 cells were co-transfected with pFLAG/B2 encoding FLAG-tagged full-length human plexin B2 and pFLAG/Rin encoding FLAG-tagged full-length human Rin. Cells co-transfected with pFLAG/B2 and pFLAG vector without insert served as negative control. IP was performed using anti-Rin antibodies. Bands marked with * represent antibodies precipitated by protein-A-agarose.

In order to determine which intracellular signaling pathways may be involved in B3 dependent neurite outgrowth, we performed yeast two-hybrid screens using the Sos-recruitment system (Figure 13). pSos fusion constructs of the intracellular parts of B3 (pSos/B3IC) and B2 (pSos/B2IC) expressed in cdc25H cells served as bait and pMyr fusion constructs of human fetal brain library cDNAs as prey molecules. Putative positive clones were used for re-transformation of cdc25H cells together with pSos/B3IC or pSos/B2IC, or pSos vector without insert. Only those clones were defined positive in which co-expression of the bait was essential in order to restore cell growth at 37°C on galactose. These experiments suggested an interaction between B3 and Rin (Ras-like protein expressed in neurons) (Figure 13, line 8), a small GTP-binding protein belonging to the Ras superfamily of GTPases. For B2, no interaction partner could be identified with this system and no interaction with Rin could be demonstrated (Figure 13, line 9). Three independent cDNAs containing the complete coding region of Rin were obtained by repeated yeast two-hybrid screens. Rin co-transfected with pSos-vector without insert was not able to induce growth on galactose at 37°C (Figure 13, line 10), showing that Rin does not activate the system unspecifically. Correspondingly, B3 and Rin could be co-immunoprecipitated from COS-7 cells transiently co-transfected with pFLAG/Rin and pIRES/B3 (Figure 14A). Rin did not co-immunprecipitate with B2 (Figure 14B).

**Figure 15:**
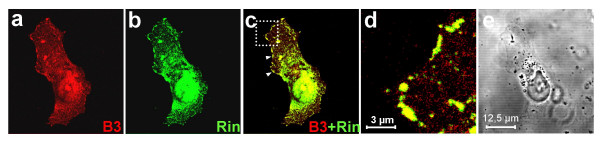
**Subcellular localization of Rin and of co-localization of Rin and B3 at the plasma membrane**. COS-7 cells were transiently transfected with pIRES/B3 encoding non-tagged full-length plexin B3 and pFLAG/Rin encoding FLAG-tagged full-length human Rin and analyzed by confocal laser scanning microscopy. **(a) **B3 was labeled using pAbB3-B and an Alexa-Fluor^®^568 (red)-conjugated secondary antibody. **(b) **Rin was labeled using anti-Flag and an Alexa-Fluor^®^488 (green)-conjugated secondary antibody. **(c) **Overlay of the images shown in a and b; yellow signals represent co-localization of B3 and Rin at the plasma membrane (arrowheads). **(d) **Magnification of the region marked in c. **(e) **Phase-contrast image of the cell shown in a-c.

COS-7 cells transiently overexpressing B3 and Rin were analyzed by confocal laser scanning microscopy (Figure 15a, b). In line with previous findings [31], Rin-specific immunofluorescence was enhanced at the plasma membrane and the nucleus of both transfected COS-7 cells (Figure 15b). Co-localization of B3 and Rin could be demonstrated at plasma membrane-associated sites (Figure 15c, d).

## Discussion

Using a neurite outgrowth assay with murine cerebellar neurons and substrate cells expressing recombinant human plexins B2 or B3, we found evidence of neurite outgrowth-promoting activity of both plexins. Up to now, in the nervous system plexins were known to act as receptors involved in repulsion and growth cone collapse. The observed stimulation of neurite outgrowth by human plexins B2 and B3 could not be explained by the mechanisms known so far for B-plexins. For *Xenopus *plexin, homophilic interaction in *trans *has been described [32]. Homophilic interaction of various neuronal transmembrane proteins, including molecules involved primarily in repulsion, has been implicated in stimulation of neurite outgrowth. Therefore, we hypothesized that homophilic interaction of plexins B2 and B3 may underlie the neurite outgrowth-promoting activity of both plexins. Using cell aggregation assays and immunoprecipitation we found evidence of human B2- and B3-specific homophilic interaction in *trans*. Furthermore, human and murine B3 co-immunoprecipitated, and we could show expression of *PlxnB3 *in cultured murine cerebellar neurons. There seemed to be a correlation between plexin-dependent cell aggregation, adhesion, and neurite outgrowth stimulation, as B3, compared to B2, had a stronger effect on all features. These data, along with the fact that homophilic interaction of various neuronal CAMs has been implicated in stimulation of neurite outgrowth [33], support the hypothesis that homophilic interaction of B3 and also possibly that of B2 is involved in stimulation of neurite outgrowth and that in the assay presented both human plexins may stimulate neurite outgrowth of murine cerebellar neurons via a quasi homophilic interaction of the human and murine homologs. However, a reverse signaling mechanism in which B3 would act as a ligand for a yet unknown receptor cannot be ruled out as an explanation for the observed B3-dependent stimulation of neurite outgrowth. Such a reverse signaling mechanism has been described for semaphorin 6D / plexin A1 in cardiac development [34].

For *plxnB3*, both glial and neuronal expression have been demonstrated [17,29,35]. Using combined *in situ *hybridization and immunocytochemistry, we found *plxnB3 *mRNA in cultured primary cerebellar neurons of six days old mice. *PlxnB3 *mRNA was also detected in adult murine cerebellum and we observed prominent neuronal B3-specific immunostaining in adult human cerebellum. Therefore, homophilic interaction of B3 is supposed to be a possible mechanism underlying the stimulation of neurite outgrowth of cerebellar neurons in the assay presented. The new ISH-probe used in this work and the probe used by Cheng et al. [35], both cover major parts of the 3'-UTR of *PlxnB3*. Probes hybridizing more upstream appear to detect a lower level of neuronal and a more pronounced non-neuronal expression of *PlxnB3 *[29,36]. We also found in addition to neuronal staining a non-neuronal staining pattern using the same probe as Worzfeld et al. [29]. These data suggest the existence of cell-type specific isoforms of B3 with different 3'-ends of the mRNA. In human organs we found evidence for the expression of such isoforms. However, in mouse EST database (NCBI dbEST) the 3'-end of *PlxnB3 *transcripts is strongly overrepresented with more upstream sequences represented very scarcely thus not allowing the rapid detailed analysis of tissue-specific expression of B3 isoforms.

B3 is a known receptor of semaphorin 5A that induces cellular collapse, growth cone collapse, has axon-repelling activity, and leads to inhibition of integrin-based adhesion of NIH-3T3 fibroblasts expressing transfected plexin B3 [17,18]. Our findings of both B3- and B2-associated cell aggregation and stimulation of neurite outgrowth suggest the possibility that plexins B2 and B3, via homophilic interaction, respectively, are also involved in signaling pathways independent of semaphorins. The sema domain of semaphorins contains a plexin interaction site [37]. By immunoprecipitation of various deletion constructs we showed that the sema domain of B3 was necessary and sufficient for the homophilic interaction. Since B3 does not co-immunoprecipitate with plexins A1, B1, or B2, and since B3-positive cells do not aggregate with L1-positive cells, the homophilic interaction seems to be highly specific. Therefore, the sema domain of B3 may be involved in both homophilic interaction and heterophilic interaction with semaphorin 5A. Under the experimental conditions presented for homophilic co-IP of recombinant B3, B3 also co-immunoprecipitates with semaphorin 5A. Since recombinant B3 and semaphorin 5A were co-expressed in the cells used for these experiments and semaphorin 5A could be co-immunoprecipitated by B3 despite its strong homophilic *trans*-interaction, both homophilic and heterophilic interactions of B3 may co-exist *in vivo*. Although the sema domain of B3 seems to be involved in both types of interaction, it is not clear whether semaphorin 5A and plexin B3 compete directly for B3 binding, whether different co-receptors are involved, and which signal transduction pathways are triggered by the different types of interaction.

B3 may be a multifunctional player in cell adhesion and both neurite outgrowth and repulsion, possibly due to competing ligands inducing "opposite" effects on neuronal morphology. There is a growing number of CAMs showing bifunctional characteristics with respect to involvement of homophilic interaction in stimulation of neurite outgrowth, neuronal attraction, migration, or axonal fasciculation, and heterophilic interactions in various functions including repulsion and growth cone collapse. A prominent example is represented by L1, known for its strong stimulation of neurite outgrowth in association with its homophilic binding [38] and also involved in semaphorin 3A mediated repulsion of cortical axons as part of a heteromultimeric receptor complex including plexin A1 and neuropilin 1 [12]. The Roundabout (Robo) receptor, a transmembrane glycoprotein sharing structural homology with a number of neuronal CAMs of the immunoglobulin (Ig) superfamily, is receptor for Slit, an extracellular matrix protein. Slit controls midline crossing of axons by inducing growth cone repulsion upon interaction with Robo [39]. On the other hand, homophilic *trans*-interaction of Robo promotes cell adhesion and neurite outgrowth [40], most likely reflecting Robo's known role in selective axon fasciculation. The majority of the known CAMs which show involvement of homophilic interaction in stimulation of neurite outgrowth or neuronal migration contain Ig (± fibronectin type III) or cadherin domains; these CAMs include L1, NCAM, Robo1, Robo2, fasciclin II, LAMP, DM-GRASP, N-cadherin, and Celsr2 [38,40-48]. Currently, the number of known CAMs with homophilic binding and neurite outgrowth stimulating characteristics is rapidly growing, with an increasing variety of molecular features not shared by Igs, fibronectins, or cadherins, as e.g. the AMIGOs or ninjurins [49-51]. Our work shows that B3 and suggests that also B2 belong to this latter group, adding further molecular heterogeneity to the group of homophilic CAMs with neurite outgrowth stimulating capacity.

Searching for intracellular pathways involved in the B2- and B3-dependent neurite outgrowth using the intracellular parts of B2 and B3 as bait in a yeast two-hybrid screen of human fetal brain cDNA we identified Rin, a neuron-specific and calmodulin-binding Ras-related GTPase, as interaction partner for B3 [52]. An interaction between B3 and Rin in mammalian cells (but not between B2 and Rin) could be shown by co-immunoprecipitation of recombinant B3 and Rin expressed in COS-7 cells in which co-localization of these proteins at plasma membrane-associated sites could be shown by confocal laser scanning microscopy. Therefore, one may assume physiological interaction of B3 and Rin as part of a neuronal receptor complex involved in B3-dependent signaling. Expression of recombinant Rin induces neurite outgrowth in rat pheochromocytoma PC12 cells and Rin interacts with the transcription factor Brn-3a, which is known to regulate different genes involved in neuronal differentiation and survival [31,53]. If the homophilic interaction of B3 is responsible for B3-dependent stimulation of neurite outgrowth one may speculate therefore that Rin may be involved in this process. Since Rin and B2 do not interact in yeast or mammalian cells, this interaction seems to be specific for B3 and other mechanisms seem to be involved in B2-dependent stimulation of neurite outgrowth. The Rin-interacting intracellular subdomain of B3 remains to be identified. This may help to elucidate how plexins may be involved in common and subtype-specific intracellular signaling pathways [8].

Further experiments are required to investigate whether homophilic interaction of B2 or B3 is responsible for the observed stimulation of neurite outgrowth, or whether these plexins function as heterophilic ligands of yet unknown neuronal receptors in a reverse signaling mechanism.

## Conclusion

Our data suggest an involvement of the homophilic interaction of plexin B3 in stimulation of neurite outgrowth. The neuron-specific small GTPase Rin, known for its neurotrophic characteristics, was identified as intracellular interaction partner of B3. Therefore, both, neuronally and non-neuronally expressed plexin B3 may be involved in semaphorin-independent signaling positively influencing neuritogenesis.

## Methods

### Analysis of the expression of full length B3 and its isoforms

For probe preparation, a *PLXNB3-*specific fragment including nucleotides 833–1,814 of AF149019 was amplified by RT-PCR and cloned into pCRII-TOPO (Invitrogen, Karlsruhe, Germany). The insert was labeled with [α^32^P]-dCTP and hybridized to normalized Multiple Tissue northern Blot and poly (A)^+ ^Multiple Tissue Expression Array (BD Biosciences). The northern blot was stripped and rehybridized with a β-actin control probe.

The distribution of three isoforms of *PLXNB3 *due to alternative splicing of the 3'- part of exon 27 was analyzed by RT-PCR form various human organs using forward primer TTCCTCCTCACG|CTCATCCACAC (splice junction marked by bar) and isoform-specific reverse primers. A 698 bp fragment containing full length exon 27 was amplified using reverse primer TCTGGGAC|CTTGTAGTGTTG. A 536 bp fragment lacking the 3'-terminal part of exon 27 and coding for a C-terminally truncated B3 was amplified using reverse primer CAGGCCTGAGCGCCACT|CTTCTC. A 356 bp fragment lacking (in-frame) 246 bp of exon 27 was amplified using reverse primer AGGCCTGAGCGCCACT|CTGTCAC.

### Plasmid constructs

pcDNA3 expression constructs encoding VSV-tagged human plexins B1 and A1 were kindly provided by Dr. L. Tamagnone. Human *PLXNB2 *cDNA (KIAA 0315) was kindly provided by Dr. T. Nagase. *PLXNB3 *cDNA [GenBank:AF149019] identified by screning fetal brain 5' Stretch Plus cDNA λ phage library (BD Biosciences) and 5'RACE and *PLXNB2 *cDNA were cloned into pIRES (BD Biosciences) and pFLAG (Sigma, Taufkirchen, Germany) vectors. The expression constructs were named correspondingly pFLAG/B2, pIRES/B3, and pFLAG/B3. Stop codon of *PLXNB3 *was changed to codon for Ser by PCR-based site directed mutagenesis. The mutant product was cloned in-frame to pEGFP-N1 (BD Biosciences) or myc-tagged pSecTag2B (Invitrogen, Karlsruhe, Germany). The constructs were named pEGFP-N1/B3 and pSecTag2B/B3. pFLAG/B3Δic, lacking intracellular part (aa 1,328–1,909) of B3, was generated by deleting a *Bam*HI/*Eco*RI fragment of pFLAG/B3. B3 deletion constructs pcDNA3.1/B3sema and HA-tagged pcDNA3.1/B3semaHA, encoding sema domain (aa 1–468) and pcDNA3.1/B3ΔsemaΔic lacking sema and intracellular (aa 1,337–1,909) domains were generated by PCR and cloned directly to pcDNA3.1D/V5-His (Invitrogen). Mouse B3 cDNA was PCR-amplified from mouse brain 5'STRETCH PLUS cDNA (BD Biosciences) and cloned to pcDNA3.1D/V5-His in order to generate pcDNA/mB3V5 encoding C-terminally V5-tagged truncated mouse plexin B3 (aa 1–1,252), lacking most of its intracellular part. Human full-length semaphorin 5A cDNA was PCR amplified from human brain QUICK-Clone cDNA and cloned into pFLAG. Human full-length Rin was PCR amplified from the pMyr construct identified in the CytoTrap^® ^system and cloned into pFLAG (pFLAG/Rin). The expression construct for L1 (pIRES/L1) was described before [28].

### Antibodies

Rabbit polyclonal antibodies (pAb) against extracellular domains of B3 were produced by immunization with peptides TSRCVTLPLDSPESYP (human sema domain, aa 354–369) for pAbB3-A, VQASRAQPQDPQPRRSC (third IPT domain of human B3, aa 1,058–1,074) for pAbB3-B, and VFRRRGARAQTEYRS (mouse B3 sema domain aa 227–241) for pAbmB3. Affinity-purified antisera were diluted 1:50 for immunocytochemistry (IC) or immunoprecipitation (IP), and 1:3,000 for Western blotting (WB). pAbex2 against human L1 has been described before [28]. Mouse monoclonal antibody (mAb) anti-Sos1 (BD Biosciences) for detection of bait constructs was used at 1:4,000 dilutions for WB. Anti-hRin mAb (ICN; Eschwege, Germany) was diluted 1:5,000 for WB and 1:50 for IC. EGFP-tagged recombinant proteins were detected by WB using mAb diluted 1:100 (Living Colors^® ^A.v. peptide anti-EGFP; BD Biosciences). Anti-c-myc mAb (Sigma) was diluted 1:500 for WB and 1:50 for IP. Anti-HA-tag rabbit pAb (BD Biosciences) was diluted 1:1,000 for WB and 1:50 for IP. Anti-FLAG mAb (Sigma), Anti-VSV-Glycoprotein mAb (Sigma) and Anti-V5 mAb were diluted for WB 1:1000, 1:10000 and 1:5000, respectively.

### Protein expression, Western blotting (WB), and immunoprecipitation (IP)

Complete mouse brain, human corpus callosum, and human neocortex samples were lysed in tissue lysis buffer (20 mM tris-HCl pH7.6, 140 mM NaCl, 5 mM EDTA, 5% glycerol, 1% NP40, 0.1% SDS, complete protease inhibitor cocktail [Roche, Mannheim, Germany]), separated under reducing conditions by SDS-PAGE, blotted onto PVDF membrane (Millipore, Eschborn, Germany), followed by detection using the antibodies described above and Immun-Star Chemiluminescent system (Bio-Rad, Munich, Germany). Lipofectamine 2000 (Invitrogen) was used for all transfections. Polyclonal cell lines stably overexpressing B3 or B2 were generated as described previously for L1 [28]. Homogeneous cell surface expression and similar total levels of expression of B3, B2, and L1 was verified by living-cell IC and WB. For IP cells were lysed 30 h post-transfection in 500 μl cell lysis buffer (20 mM tris-HCl pH7.5, 150 mM NaCl, 1% TritonX-100, complete protease inhibitor cocktail) and centrifuged (10 min, 12,000 rpm). Supernatants were incubated (2 h, 4°C) with pAbB3-B, anti-myc, anti-FLAG (Sigma), or anti-HA (BD Biosciences) and precipitated (12 h, 4°C) by protein-A agarose (Roche). After washing once with cell lysis buffer and twice with washing buffer (20 mM tris-HCl pH 7.5, 500 mM NaCl and 0,1% TritonX-100), bound protein was eluted by boiling in SDS-PAGE gel loading buffer, and immunodetected as described for WB.

### Glycosylation analysis and surface protein biotinylation

One day after transfection COS-7 cells were treated with 10 μg/ml glycosylation inhibitor tunicamycin (Sigma) for 24 h prior to lysis. Alternatively, lysates of untreated cells were incubated (12 h, 37°C) with 750 U endo-β-N-acetylglucosaminidase H (Endo H; New England Biolabs, Frankfurt, Germany). For surface biotinylation adherent cells were washed three times with PBS at 4°C and incubated in 0.5 mg EZ-Link^® ^Sulfo-NHS-LC-Biotin (Pierce, Bonn, Germany) /ml PBS (30 min) on ice. After washing three times in PBS (4°C) cells were lysed in 1 ml ice-cold lysis buffer and centrifuged (10 min, 4°C, 12,000 rpm). The supernatant was incubated (2 h, 4°C) with antibodies pAbB3-B or anti-FLAG, and precipitated (12 h, 4°C) with Protein A-agarose. After washing twice with washing buffer bound protein was eluted. PAGE, blotting, and detection were done as described above. Biotinylated protein was detected by alkaline phosphatase-conjugated streptavidin (Sigma).

### Immunocytochemistry (IC), in situ hybridization (ISH) and immunohistochemistry (IHC)

To detect cell surface expression of B3, IC was performed on living COS-7 and NIH-3T3 cells stably transfected with pIRES/B3 using pAbB3-B and Cy3-conjugated secondary antibodies. For detection of Rin expression and co-localization experiments cells transfected with pFLAG/Rin or co-transfected with pFLAG/Rin and pIRES/B3 were fixed with 4% paraformaldehyde (PFA) in PBS before incubation with anti-Rin and pAbB3-B and Alexa-Fluor^®^568- or -488-conjugated secondary antibodies and analyzed by confocal laser scanning microscopy.

For ISH, two different digoxigenin-labeled riboprobes were synthesized. The probes correspond to nucleotides 4,647 – 5,936 and 3,744 – 5,679 [29] of *PlxnB3 *cDNA [GenBank:NM_019587]. After fixation (4% PFA) and acetylation slides were hybridized (12 h, 55°C), followed by washing twice with 0.2 × SSC (20 min, 55°C), three times with 0.2 × SSC in 50% formamide (60 min, 55°C), once with 0.2 × SSC (10 min, RT), and final equilibration in TBS (10 min). Colorimetric immunodetection (NBT/BCIP) was performed according to the manufacturer's instructions (Roche).

To analyze the expression of *PlxnB3 *in various brain cell types, astrocytes, oligodendrocytes, and neurons of mechanically dissociated cerebellum of six days old mice were grown under cell type specific selective conditions according to the protocols described by [54] and used for combined ISH and IC. ISH was done as described before using a digoxigenin-labeled riboprobe corresponding to nucleotides 4,647–5,936 of *PlxnB3 *cDNA. After hybridization slides were washed twice with 0.2 × SSC (20 min, 55°C) and three times with 0.2 × SSC in 50% formamide (20 min, 55°C). For IC slides were equilibrated in PBS, incubated (30 min) in blocking solution (Roche), followed by incubation (12 h, 4°C) with mAb against Neuromodulin (Transduction Laboratories), NeuN (1:25; Chemicon), GFAP (1:100; Transduction Laboratories, Heidelberg, Germany) or CNPase (Sigma), washed three times in PBS, followed by detection (45 min, RT) using Cy3-labeled rabbit-anti-mouse antibody (1:200; Dianova, Hamburg, Germany), washing with PBS /0.1% Tween, and mounting in Mowiol (Calbiochem).

For IHC formaldehyde-fixed and paraffin-embedded slices (7 μm) of post mortem human brain (post mortem time 47 h) were dewaxed, hydrated and further fixed in 4% PFA. Endogenous peroxidase was quenched by 3% H_2_O_2 _(30 min). The slides were treated with 6 M urea /0.1 M glycin (pH3.5), blocked (2% BSA, 3% goat serum, 0.2% TritonX-100 in PBS), and incubated with pAbB3-B (1:50; 12 h, 4°C) detected using Vectastain ABC Kit (Vector Laboratories), DAB chromogen, and immunoperoxidase reaction. The slides were counterstained with hematoxylin.

All animal experiments have been approved by the local government body regulating animal research. The human brain tissue used in the experiments was obtained from the officially approved local research brain bank.

### Neurite outgrowth and cell aggregation assays

NIH-3T3 cells were stably transfected in order to express recombinant *L1CAM*, *PLXNB2*, and *PLXNB3 *and used for cell aggregation assays and as substrate cells for primary murine cerebellar neurons in neurite outgrowth assay performed as described previously [28]. Cell aggregation assays were performed essentially according to the method of [30]. Monolayers of substrate cells were incubated with 2 mM EDTA in PBS (15 min, RT), dispersed by pipetting, diluted to 1 × 10^6 ^cells/ml (N_0_) in DMEM or Hanks' balanced salt solution (HBSS) ± 1 mM Ca^2+ ^and 0.5 mM Mg^2+^, and incubated at 37°C. N_t_/N_0_, representing aggregation-dependent decrease of total particle number at incubation time t was determined in aliquots taken every 20 min out of the suspension immediately after mixing the suspension by gentle inversion. To determine the molecular specificity of cell aggregation, control and transfected cells were stained by lipophilic dyes DiO and DiI, respectively (Molecular Probes, Leiden, The Netherlands) using 5 μl dye solution /ml (2 h, 37°C). DiO- and DiI-stained cells were diluted in DMEM to 5 × 10^5 ^cells/ml, mixed 1:1, incubated (45 min, 37°C), spotted on coverslips, fixed (4% PFA; 10 min), and washed in PBS (10 min) prior to fluorescence microscopy.

### Yeast two-hybrid analysis

To identify intracellular interaction partners of plexins B2 and B3 we used the CytoTrap^® ^yeast two-hybrid system (Stratagene) also known as Sos Recruitment System [55]. This system is based on the recruitment of human Sos (hSos) to the plasma membrane in the mutant temperature-sensitive yeast strain cdc25H. This strain is unable to grow at the restrictive temperature of 37°C unless activation occurs through translocation of hSos to the plasma membrane via interaction between two-hybrid proteins. The intracellular parts of B2 (bp 3,960–5,840) and B3 (bp 3,840–5,680) were amplified by PCR using primers with *Sal*I and *Not*I restriction sites and cloned into *Sal*I/*Not*I-cleaved pSos vector in order to create hSos fusion constructs as bait for the CytoTrap system (pSos/B2IC and pSos/B3IC). We screened a human fetal brain plasmid cDNA library with an average insert size of 1.3 kb in the pMyr expression vector (Stratagene) according to the manufacturer's instructions. Conventional yeast transformation by the lithium acetate method was used. Cdc25H cells were co-transformed with pSos/B2IC or pSos/B3IC and pYES/mGAP in order to reduce isolation of Ras GTPase false positive clones [56]. Expression of bait constructs was confirmed by immunoblotting of yeast lysates with an anti-hSos1 antibody. These pre-transformed cdc25H strains were transformed with pMyr-cDNA library plasmids and incubated at 22°C for 5 days on selective minimal glucose plates lacking leucin, uracil, and tryptophan. A total of ~2.5 × 10^6 ^transformants were replica-plated onto selective minimal galactose plates and grown up (7 days, 37°C). From a first selection of 350 clones 39 putative positive clones were isolated after a second round of selection on galactose plates at 37°C. Bait-prey protein interactions of putative positive clones were analyzed by re-transformation of the cdc25H yeast strain with both the respective prey-encoding pMyr-plasmid together with pSos/B2IC, pSos/B3IC, or pSos vector without insert.

## Authors' contributions

AV and UF conceived of the study and participated in its design and coordination; UF wrote the grant application. AV participated in cloning and supervised some of the experiments. UF, AV, and CH drafted the manuscript. CH participated in cloning and carried out all experiments except *in situ *hybridization, immunohistochemistry, northern blot and RT-PCR that were done by SK. GR participated in yeast two hybrid screening and confocal laser microscopy. All authors read and approved the final manuscript.
